# Isolation and Characterization of Intestinal Epithelial Cells from Normal and SIV-Infected Rhesus Macaques

**DOI:** 10.1371/journal.pone.0030247

**Published:** 2012-01-26

**Authors:** Diganta Pan, Arpita Das, David Liu, Ronald S. Veazey, Bapi Pahar

**Affiliations:** 1 Division of Comparative Pathology, Tulane National Primate Research Center, Covington, Louisiana, United States of America; 2 Division of Microbiology, Tulane National Primate Research Center, Covington, Louisiana, United States of America; 3 Tulane University School of Medicine, New Orleans, Louisiana, United States of America; University of Montreal, Canada

## Abstract

Impairment of intestinal epithelial barriers contributes to the progression of HIV/SIV infection and leads to generalized HIV-induced immune-cell activation during chronic infection. Rhesus macaques are the major animal model for studying HIV pathogenesis. However, detailed characterization of isolated rhesus epithelial cells (ECs) from intestinal tissues is not well defined. It is also not well documented whether isolated ECs had any other cell contaminants from intestinal tissues during the time of processing that might hamper interpretation of EC preparations or cultures. In this study, we identify and characterize ECs based on flow cytometry and immunohistochemistry methods using various enzymatic and mechanical isolation techniques to enrich ECs from intestinal tissues. This study shows that normal healthy ECs differentially express HLA-DR, CD23, CD27, CD90, CD95 and IL-10R markers. Early apoptosis and upregulation of ICAM-1 and HLA-DR in intestinal ECs are thought to be the key features in SIV mediated enteropathy. The data suggest that intestinal ECs might be playing an important role in mucosal immune responses by regulating the expression of different important regulatory and adhesion molecules and their function.

## Introduction

The intestinal mucosal immune response in healthy individuals is characterized by a balance between immunity, which protects mucosal surfaces from harmful microbes, and tolerance, which permits the intestinal mucosa to interact in a nonpathogenic way with the commensal bacteria and dietary antigens to which it is constantly exposed [1]–[3]. The small and large intestinal epithelium is simple columnar, non-ciliated cells. Certain epithelial cells (ECs) lining the small intestine also had the function to absorb nutrients from the digestion of food. In glands, ECs are specialized to secrete specific chemical substances such as enzymes, hormones and lubricating fluids. HIV-1 infection is initiated primarily on the mucosal surfaces, through sexual transmission [1], [4]. The epithelial layer seems to be an efficient mechanical barrier against several pathogens including HIV-1 [5]. However, mucosal transmission accounts for more than 90% of HIV infections [6]–[8]. Intestinal ECs preferentially express coreceptor molecules like CCR5 rather than CXCR4, however, they generally do not express the HIV-1 receptor CD4 [8]. Moreover, it is believed that for an efficient HIV-1/SIV infection, the virus needs to bypass the epithelial barrier to enter in the intraepithelial lymphocytes (IEL) or lamina propria lymphocytes (LPL). The primary ECs were able to transfer CCR5 tropic virus more efficiently than CXCR4 tropic virus through transcytosis to indicator cells by *in vitro* experiments [9]–[11]. Recent studies have shown that mucosal EC respond directly to HIV envelope glycoproteins by upregulating inflammatory cytokines that lead to impairment of barrier functions [12]. The majority of studies on ECs and HIV interaction have been performed using primary EC cultures from intestinal and reproductive tissues or cell lines *in vitro* [12], [13]. However, detailed isolation and characterization of rhesus ECs from intestinal tissues are poorly documented, whereas the rhesus macaque (RM) model is well recognized for understanding HIV/SIV pathogenesis, disease progression and HIV vaccine development [14]. It is also not well documented whether isolated ECs have other cell contaminants from intestinal tissues during the time of processing or whether isolation methods could be improved or optimized to reduce contamination that might hamper the study design using EC cultures. Moreover, these ECs in normal uninfected and SIV infected RMs were not characterized with respect to memory and/or effector status, adhesion, antigen presentation, or regulatory receptor expression compared to intestinal CD45+ leukocytes.

Here we identify and characterize ECs using flow cytometry and immunohistochemistry methods where we have compared various enzymatic and mechanical isolation techniques to enrich ECs from intestinal tissues. This study shows that ECs are positive for HLA-DR, CD23, CD27, CD90, CD95 and IL-10R phenotypes. Early apoptosis and upregulation of ICAM-1 and HLA-DR in intestinal ECs are thought to be the key features in SIV mediated enteropathy. The data suggest that intestinal ECs might be playing an important role in mucosal immune responses by regulating the expression of different important regulatory and adhesion molecules and their function.

## Results

### Increased epithelial cell isolation by either DTT or EDTA treatment

**Figure 1:**
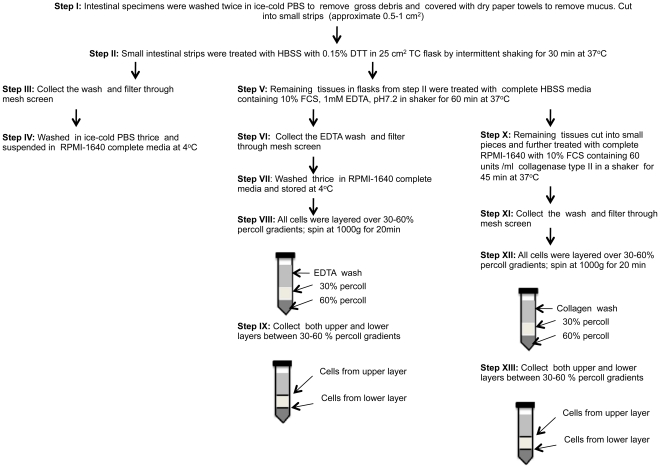
Schematic representation of epithelial cell and leukocyte isolation protocols from intestinal tissues after different enzymatic treatments. Note that this protocol explains cell isolation procedures with initial DTT treatment.

**Figure 2:**
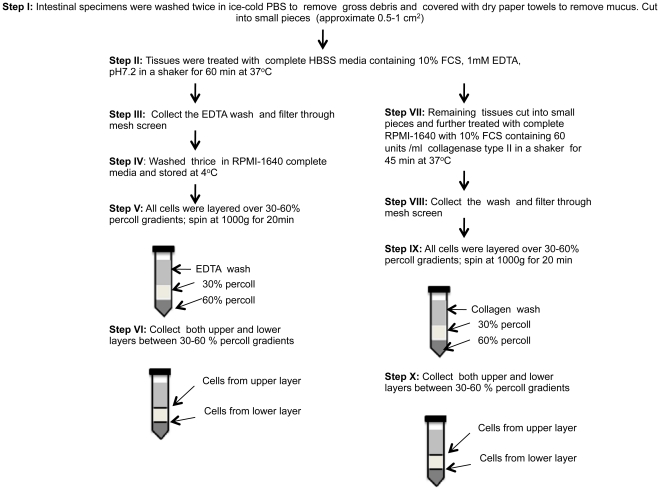
Schematic representation of epithelial cell and leukocyte isolation protocols from intestinal tissues after different enzymatic treatments. Note that this protocol explains cell isolation procedures with initial EDTA treatment. No prior DTT treatment has been used in this protocol.

**Figure 3:**
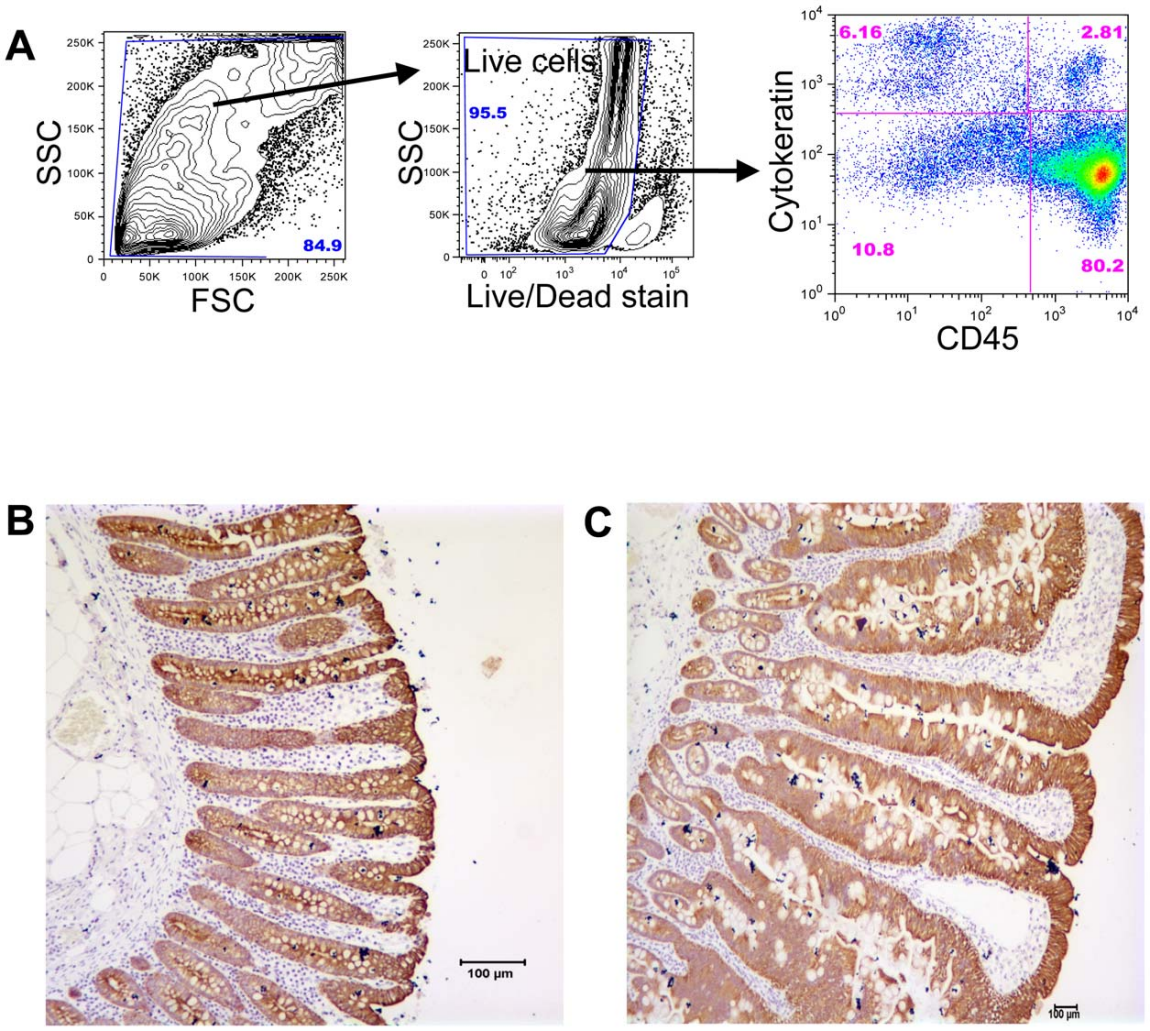
Phenotyping epithelial cells of normal rhesus macaques. (**A**) Representative expression of cytokeratin (CK; epithelial cell marker) and CD45 (leukocyte marker) in jejunum intestinal cells isolated from lower layer (“lymphocyte enriched”) of percoll gradient after treating with DTT and EDTA solution (Fig. 1; Step IX). Note that 2.8% of cells were double positive for CK and CD45 receptors. Fractions of double negative CK−CD45− cells were also evident from isolated cells. Live cells were gated first from all acquired cells and plotted based on CK and CD45 markers. Visualization of epithelial cells both in (**B**) colon at 20× and (**C**) jejunum at 5× resolution were detected by immunohistochemistry staining using anti-CK monoclonal antibody with hematoxylin counterstain.

**Figure 4:**
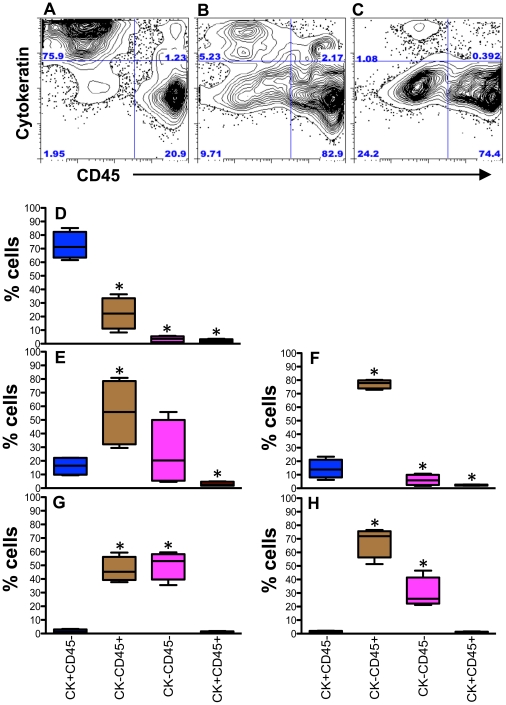
Isolation of epithelial cells and leukocytes from intestinal tissues with DTT, EDTA and collagenase treatments. Representative dot plots of total cells from (**A**) DTT wash (Fig. 1, step IV); (**B**) lower layer of percoll density gradient, isolated after EDTA treatment (Fig. 1, step IX); (**C**) lower layers of percoll density gradient, isolated after collagenase treatment (Fig. 1, step XIII) from jejunum tissue showing distribution of epithelial cells (ECs; cytokeratin as an epithelial cell marker) and CD45 leukocytes in a normal uninfected healthy rhesus macaque. Each quadrant shows percentages of specified cell populations. Note increased percentage of ECs were isolated from DTT wash compared to other methods examined. Mean frequencies of cytokeratin (CK) positive, CD45 positive, double positive and double negative for CK and CD45 cell subsets are shown as box and whisker vertical bars for different isolation protocols as specified in Fig. 1. In summary all the specified bars represent (**D**) cells isolated from DTT wash (Fig. 1, step IV); (**E**) upper layer cells from percoll density gradient isolated after EDTA treatment (Fig. 1, step IX); (**F**) lower layer cells from percoll density gradient isolated after EDTA treatment (Fig. 1, step IX); (**G**) upper layer cells from percoll density gradient isolated after collagenase treatment (Fig. 1, step XIII); and (**H**) lower layer cells from percoll density gradient isolated after collagenase treatment (Fig. 1, step XIII) from jejunum in healthy, normal, uninfected rhesus macaques (n = 5). Note that in all isolation protocols, there was a variable amount of CD45+ and double negative CK−CD45− cells contamination observed. * Indicates significant differences between CK+CD45− and other different subsets of total cells within the specified isolation protocol.

**Figure 5:**
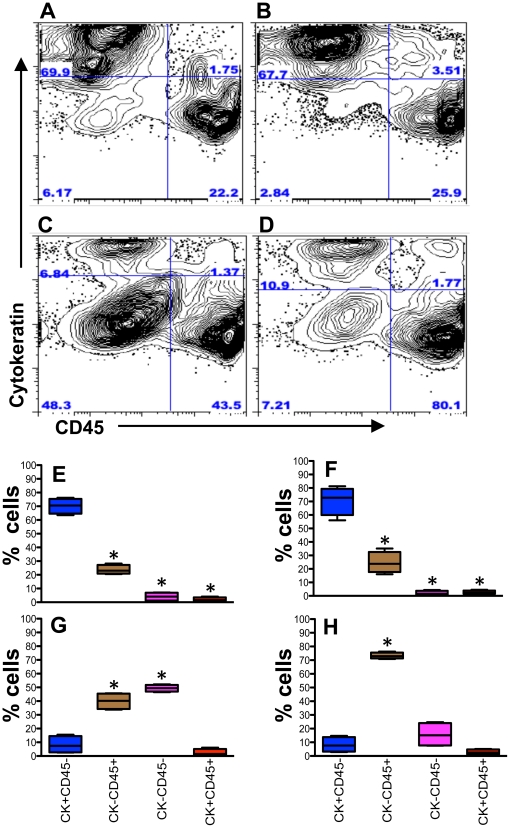
Isolation of epithelial cells and leukocytes from intestinal tissues with EDTA and collagenase treatment. Representative dot plots of total cells from (**A**) upper layer of percoll density gradient isolated after EDTA treatment (Fig. 2, step VI); (**B**) lower layer of percoll density gradient isolated after EDTA treatment (Fig. 2, step VI); (**C**) upper layer of percoll density gradient isolated after collagenase treatment (Fig. 2, step X); and (**D**) lower layer of percoll density gradient isolated after collagenase treatment (Fig. 2, step X); in jejunum tissue showing distribution of epithelial cells (ECs; cytokeratin as an epithelial cell marker) and CD45 leukocytes in a normal uninfected healthy rhesus macaque. Each quadrant shows percentages of specified populations. Note increased percentage of ECs were isolated from both upper and lower layer cells of percoll density gradient isolated after EDTA treatment compared to other methods examined. Mean frequencies of cytokeratin (CK) positive, CD45 positive, double positive and double negative for CK and CD45 cell subsets are shown as box and whisker vertical bars for different isolation protocols as specified in Fig. 2. In summary all the specified bars represent (**E**) upper layer cells from percoll density gradient isolated after EDTA treatment (Fig. 2, step VI); (**F**) lower layer cells from percoll density gradient isolated after EDTA treatment (Fig. 2, step VI); (**G**) upper layer cells from percoll density gradient isolated after collagenase treatment (Fig. 2, step X); and (**H**) lower layer cells from percoll density gradient isolated after collagenase treatment (Fig. 2, step X); from jejunum in healthy, normal, uninfected rhesus macaques (n = 5). Note that in all isolation protocols, there was a variable amount of CD45+ and double negative CK−CD45− cells contamination observed. * Indicates significant differences between CK+CD45− and other different subsets of total cells within the specified isolation protocol.

**Figure 6:**
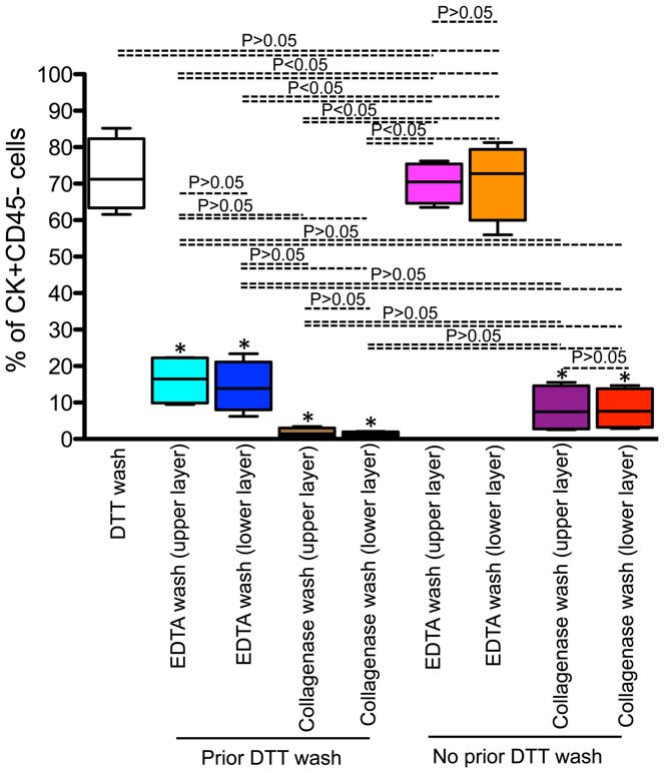
Percentages of epithelial cells retrieved from different isolation protocols. Mean percentages (± standard deviation) of isolated cytokeratin (CK)+ CD45− cells from jejunum either after DTT, EDTA or collagenase treatment with or without prior DTT wash (as discussed in details in Figs. 1 & 2) are shown from uninfected normal healthy rhesus macaques (n = 5). Note that there was increased yield of ECs either by DTT or EDTA only treatment compared to other protocols followed in this experiment. Statistical significant differences between each group of cells are shown. * Indicates significant differences in ECs isolated from either DTT or EDTA only treatment compared to other group of cells.

Epithelial cells from jejunum and colon were isolated using several enzymatic and isolation techniques that have been diagrammatically represented in Figs. 1 & 2. To characterize ECs, anti-cytokeratin and Ber-EP4 (epithelial antigen) monoclonal antibodies (MAbs) were used in both immunohistochemistry and flow cytometry assays (Fig. 3). Both anti-cytokeratin and Ber-EP4 MAbs yield similar percentages of ECs from each sample as detected by flow cytometry (data not shown). Ber-EP4 has been reported as an important epithelial cell marker to define ECs and to differentiate from mesothelial cells [15]–[20]. Epithelial cells were also identified in jejunum and colon mucosa based on their morphology, epithelial location, and immunophenotype. To identify and characterize ECs from different cell isolation protocols, we performed flow cytometry using Ber-EP4 (CK; for epithelial cell detection) and CD45 MAbs (reacts with leukocyte common antigens present in all leukocytes including lymphocytes, monocytes, granulocytes, eosinophils and thymocytes). All cells were categorized into 4 different cell subsets based on their CK and CD45 expression (CK+CD45−, CK−CD45+, CK−CD45−, and CK+CD45+). The dead cells were excluded by live/dead staining for all ECs and CD45 leukocytes analysis. Both DTT (reducing agent) and EDTA (cation chelant) solution has been used for the isolation of intestinal ECs [21], [22]. However, the purity of EC from each isolation technique has not been clearly defined, where the characterization of ECs was based on low to moderately sensitive immunohistochemistry techniques. DTT treatment only yields more purified single positive EC (CK+CD45−; 72.3%±9.9%) compared to other steps examined in our protocol (Figs. 1 & 4). Similar percentages of single positive ECs were also evident in EDTA treated upper (70.2%±5.6%) and lower (70.7%±10.6%) layer cells isolated from percoll gradient where no prior DTT treatment was followed (Figs. 2, 5 & 6). ECs isolated from upper and lower layer of EDTA wash with prior DTT treatment were 16.2% and 14.4% respectively. There was no statistical differences in the purity of ECs isolated from intestinal tissues after DTT wash and steps where EDTA was used at the beginning without prior DTT treatment (Fig. 6). ECs isolated from upper and lower layers of collagenase wash without any prior DTT wash were 8.2% and 8.2% respectively (Fig. 6). However in all of our steps in the specified protocols, there was a variable amount (1.4%–16.2%) of ECs contamination in our LPL and IEL isolation protocols (Figs. 3, 4, 5 & 6). Similarly, single positive CD45 cells were also present in all ECs isolation protocols (Figs. 3, 4 & 5). Double positive (CK+CD45+) and double negative (CK−CD45−) cells were present at varied frequencies with mean value range from 0.9%–3.0% and 3.4%–50.3% respectively in all different isolation protocols. None of our experimental procedures are able to yield 100% purified intestinal ECs population and are suggestive of single cell sorting experiment for isolating purified ECs.

### Cytokeratin expression by intestinal epithelial cells in normal rhesus macaques

**Figure 7:**
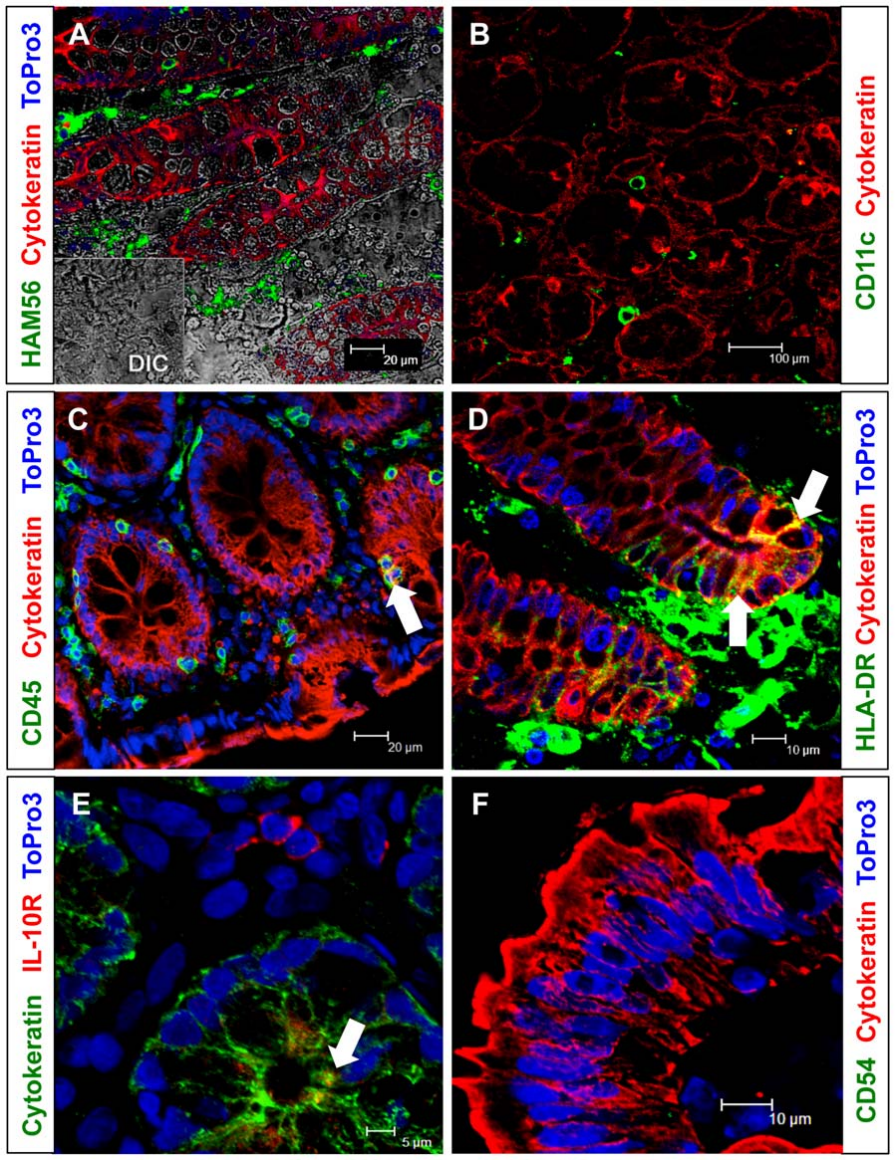
Phenotyping colon and jejunum epithelial cells (ECs) by confocal microscopy. Essentially all colon (**A–E**) ECs (cytokeratin positive) were negative for (**A**) Ham56 (macrophage marker), and (**B**) CD11c (dendritic cell marker). Very few colon ECs were double positive for (**C**) both CD45 (leukocyte marker) and cytokeratin expression. A major population of colon ECs was positive for (**D**) HLA-DR expression. However, very few colon ECs were positive for (**E**) IL-10R expression which was evident in apical regions. Jejunum ECs were also negative for (**F**) CD54 (ICAM-1, cell adhesion marker). White arrow denotes the presence of double positive cells (cytokeratin and CD45/HLA-DR/IL-10R) for the specified sample and fluorochrome.

There was no colocalization either between epithelial (cytokeratin) and Ham56 (macrophage marker), epithelial and CD11c (dendritic cell marker), and epithelial and CD54 (cell adhesion molecule) (Figs. 7A–B & 7F). Few double positive CK+CD45+ cells were evident in ECs that also support our flow cytometry data (Figs. 3 & 7C). However, increased HLA-DR expression was observed both in ECs as well as intraepithelial region (Fig. 7D). Limited IL-10R expression was also detected in intestinal ECs (Fig. 7E). In summary, ECs do not express macrophage or dendritic cell markers and they differentially express different regulatory and activation molecules.

### Immunophenotypic characterization of epithelial cells in normal intestinal mucosa

**Figure 8:**
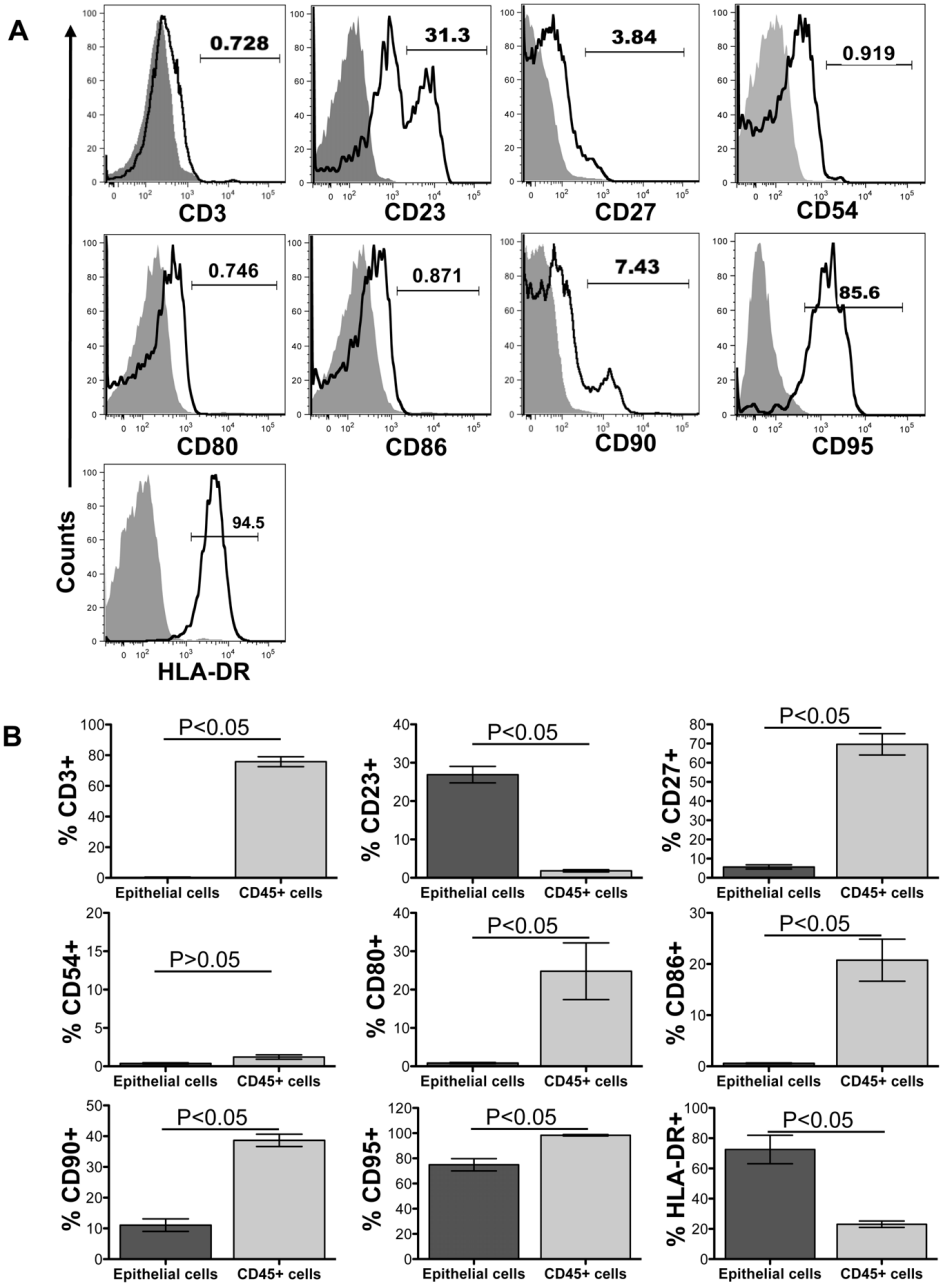
Relative expression of CD3, CD23, CD27, CD54, CD80, CD86, CD90, CD95 and HLA-DR in intestinal epithelial cells (ECs) from normal healthy macaques. (**A**) Jejunum ECs (open histogram) and isotype control (filled histogram) shown for expression of CD3, CD23, CD27, CD54, CD80, CD86, CD90, CD95 and HLA-DR using anti-CD3, anti-CD23, anti-CD27, anti-CD54, anti-CD80, anti-CD86, anti-CD90, anti-CD95 and anti-HLA-DR monoclonal antibodies respectively from a normal healthy rhesus macaque. (**B**) Mean frequencies (± standard deviation) of cell adhesion and regulatory markers are shown as bars for jejunum ECs and CD45+ leukocytes from uninfected normal rhesus macaques (n = 6). Note that there was an increased expression of CD23 and HLA-DR in ECs compared to CD45+ leukocytes from jejunum tissues. DTT washed live cells from jejunum tissue were gated from all acquired samples. All live cells were further gated based on CD45 and cytokeratin (CK) expression. Only CK+CD45− and CK-CD45+ cells were identified for further phenotypic analysis. Statistical significant differences between each group of cells are shown.

To confirm the observations detected by immunohistochemistry, single-cell suspensions of isolated cells were generated from intestinal mucosa followed by DTT wash as described in the materials and methods section. These cell suspensions were subsequently analyzed by multicolor flow cytometry analysis. In addition to lymphocytes, macrophages, monocytes and other leukocytes, these preparations also contain additional resident cells. In these preparations, ECs are identified as Ber-EP4 positive cells with no CD45 surface expression (Fig. 3). Fibroblast/myofibroblast cells were identified using CD90 (Thy-1) marker and those cells were negative for both Ber-EP4 and CD45 surface marker [23]. CD90 and α smooth muscle actin fiber has also been used for defining myofibroblast markers in human colonic mucosa [24]. Interestingly, significantly increased HLA-DR expression was observed on ECs (mean value 72.5%±23.05%) compared to CD45+ leukocytes (mean value 23.07%±5.15%) (Fig. 8). Increased expression of HLA-DR in human intestinal ECs was confirmed using a sensitive avidin biotin-peroxidase technique, which may help to explain the unique properties of intestinal ECs as antigen-presenting cells [25].

CD23, the low-affinity IgE receptor is widely distributed on B cells in the follicular mantle, on resting B cells. CD23 is also detected in monocytes and eosinophils and has influence on cell differentiation and growth of both B and T cells. CD23 expression in jejunum ECs (mean value 26.9%±3.73%) was significantly higher than CD23 expressed by CD45 leukocytes (mean value 1.81%±0.53%), however their function is not well understood (Fig. 8).

Surface receptor CD27, a type 1 glycoprotein and a member of the tumor necrosis factor receptor family was shown on a subset of B and T cells and it was thought that their expression may be required for the generation of memory and long term maintenance of B and T cell immunity [26], [27]. Interestingly ECs are also able to express CD27 markers to some extent (mean; 5.6%±2.24%; Fig. 8), however their function in ECs are also not well defined.

Intracellular adhesion molecule-1 (ICAM-1; CD54) has an important role to play in the intestinal immune response [28]–[30]. CD80 (B7.1) is a costimulatory molecule involved in T cell activation and survival. Similar to CD80, CD86 is a protein expressed on antigen-presenting cells that provide costimulatory signals necessary for T cell activation and survival. We were unable to detect any CD54, CD80 and/or CD86 expression in ECs from normal jejunum and colon intestinal tissues both by immunohistochemistry (Fig. 7) and flow cytometry (Fig. 8). Intestinal CD45+ leukocytes had significantly higher expression of CD80 (mean; 24.78%±16.52%; Fig. 8) and CD86 (mean; 20.75%±10.08%; Fig. 8) compared to ECs. However, CD45+ leukocytes had low CD54 expression as observed in ECs.

CD90, a glycophosphatidylinositol-linked glycoprotein was initially described as a differentiation marker expressed predominantly in the mouse brain and thymus [31]. The exact mechanism and roles of Thy-1 remained unanswered and it has been proposed that this molecule is involved in cell-cell interaction [32]. Recent studies in murine Thy-1 expression, its signaling properties, and the stimulatory effect of Thy-1 cross-linking on mouse T cells indicate that Thy-1 is more than just a T cell marker [33]. In humans, Thy-1 is expressed on the surface of a number of cells including neurons, retinal ganglion cells, thymocytes, vesicular pericytes, subsets of fibroblasts, activated endothelial cells, mesangial cells and mesenchymal and hematopoietic cells [23], [34], [35]. The expression of CD90 in intestinal ECs is not yet well studied. In this study, we have shown about 11.05%±6.46% of EC express CD90 (Fig. 8). In contrast, intestinal CD45+ leukocytes express significantly higher amount of CD90 (mean value 38.65%±4.88%; Fig. 8).

CD95 is a member of the tumor necrosis factor family and induces apoptosis when cross-linked by its natural ligand, CD95L. High percentages of ECs express CD95 (mean 74.9%±12.9%). However CD95 expression on ECs is significantly lower compared to intestinal CD45+ leukocytes (mean 98.36%±1.55%; Fig. 8).

**Figure 9:**
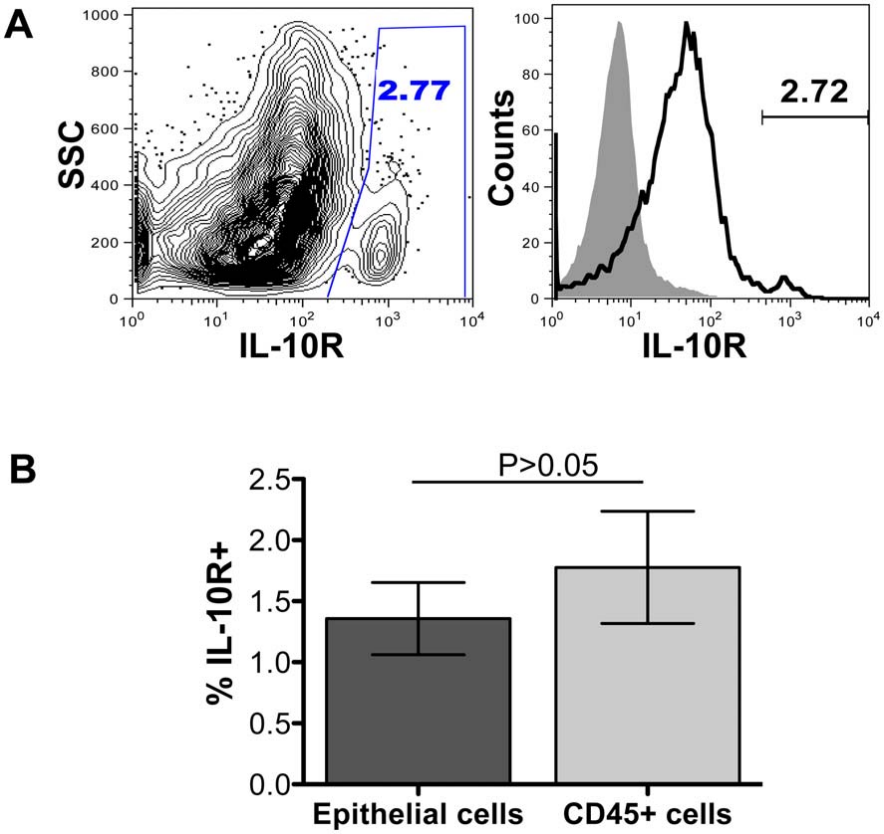
IL-10R expression in intestinal epithelial cells (ECs). (**A**) Left contour plot shows the IL-10R positive cells in the gated region from a normal healthy jejunum ECs. Jejunum ECs (open histogram) and isotype control (filled histogram) were also shown as histograms for expression of anti-IL-10R in the right panel. (**B**) Mean percentages (± standard deviation) of surface IL-10R expression are shown for both jejunum ECs and CD45+ leukocytes isolated from normal healthy rhesus macaques (n = 5). Statistical significant differences between each group of cells are shown.

IL-10 has been shown to mediate anti-inflammatory activity in cells of different lineages by interacting with IL-10R expressed on the cells. To determine the expression of IL-10R both in jejunum and colon ECs we have used anti-IL-10R MAb. Quantitative analysis by flow cytometry has shown surface IL-10R expression in ECs (mean value 1.36%±0.94% and 1.5%±0.25% for jejunum and colon respectively; Fig. 9). There was no statistically significant difference in IL-10R expression between CD45+ leukocytes and ECs (Fig. 9). In summary intestinal ECs did not express CD11c, Ham56, CD3, CD54, CD80, and CD86 receptors by either confocal or flow cytometry assays. A moderate to higher expression of CD23, CD27, CD90, CD95 and HLA-DR was detected in intestinal ECs. However, the percentages of CD23 and HLA-DR expression in ECs were higher compared to CD45+ intestinal leukocytes.

### Upregulation of apoptosis and ICAM-1 expression in intestinal epithelial cells during SIV infection

**Figure 10:**
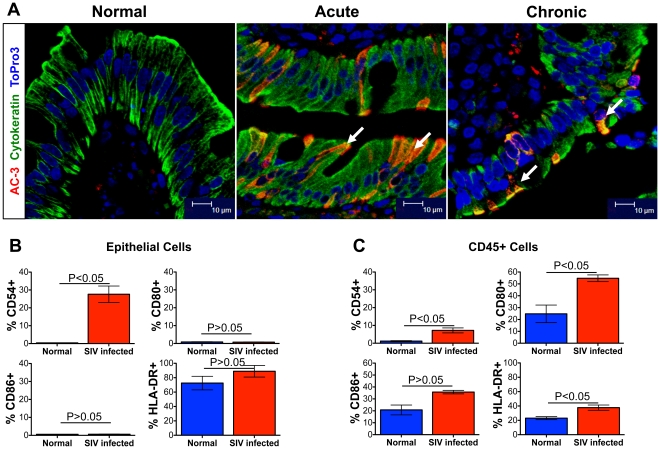
SIV infection induces apoptosis and upregulation of CD54 expression on intestinal epithelial cells (ECs). (**A**) Epithelial cells apoptosis was detected in jejunum by multi-labeled immunofluorescent confocal microscopy. Note that increased apoptosis of jejunum ECs was detected during acute (AV85; 21 days post infection) and chronic (HG58; 288 days post infection) SIV infection (as indicated by the white arrows). (**B**) Mean frequencies (± standard deviation) of surface CD54, CD80, CD86 and HLA-DR expression are shown for both jejunum ECs and CD45+ leukocytes from normal (n = 6) and chronically SIV-infected (n = 4) macaques. Note that a significant increase in CD54 expression on jejunum ECs was detected in SIV-infected macaques. However, increased expression of CD54, CD80 and HLA-DR on CD45+ leukocytes was observed in SIV-infected RMs. Statistically significant differences between each group of cells are shown.

To assess early jejunum ECs apoptosis in SIV infection, frozen archived jejunum tissues from acute (21 days post infection) and chronically (288 days post infection) SIV infected RMs were stained with anti-active caspase-3 (AC-3) antibodies. Confirmation of ECs apoptosis was performed using anti-cytokeratin antibodies in all acute and chronically SIV infected RMs (Fig. 10A). As predicted, there was an increased AC−3+ jejunum ECs in both acute and chronically SIV infected RMs suggestive of early ECs apoptosis in SIV infected RMs. To understand the role of different regulatory (CD54, CD80 and CD86) and activation (HLA-DR) molecules, expression of CD54, CD80, CD86 and HLA-DR were measured in freshly isolated ECs and CD45+ cells from chronically SIV-infected animals and compared with SIV uninfected healthy control macaques. A significant increase of CD54 expression was detected both on ECs (mean 25.8% vs 0.4%) and CD45+ (mean 7.2% vs 1.2%) cells in SIV infected RMs compared to SIV uninfected controls ECs and CD45+ cell population (Fig. 10B). Increased HLA-DR expression was also detected both in ECs (mean 88.9% vs 72.5%) and CD45+ (mean 37.7% vs 23.1%) cells from SIV infected compared to control animals. However, the difference in HLA-DR expression in ECs from SIV-infected macaques compared to uninfected control RMs was not statistically significant. No significant differences in CD80 and CD86 expression were detected in normal uninfected and SIV-infected RMs. In contrast, a significantly higher expression of CD80 (mean 54.7% vs 24.8%) was detected in CD45+ cells from SIV-infected RMs compared to uninfected controls (Fig. 10B). CD86 expression was higher on intestinal CD45+ cells (33.2% vs 20.7%) in SIV-infected animals, however the difference was not statistically significant between control RMs (Fig. 10B). In summary, increased apoptosis and upregulation of ICAM-1 and HLA-DR expression in intestinal ECs from SIV infected RMs are suggestive of its role in SIV enteropathy.

## Discussion

Intestinal epithelium primarily takes part in the digestive system. However, they play an important part in the immune system, both as a barrier and as a first-line pathogen recognition system [36]. The present study was designed to identify and characterize intestinal ECs and their surface receptors for better understanding their functional properties and immune protection both in normal and SIV-infected RMs.

Combinations of mechanical preparation and enzymatic digestion methods to enrich RM intestinal ECs from tissues obtained from necropsied animals were compared. Using these techniques, large numbers of intestinal ECs, with a viability of 90–96%, were obtained. Bacterial contamination is rarely a problem in these isolation procedures, as the majority or bacterial flora are removed by several washing steps and percoll separation. Cells isolated from directly after DTT wash may contain more bacterial contamination that needs further investigation. The use of DTT or EDTA was found to be best for isolating intestinal ECs as the isolation procedure is faster, yields increased percentages of ECs, and takes fewer steps to identify ECs. Further collagenase enzymatic treatment reduces the yield of ECs and increases the percentages of leukocytes. Overall in all isolation protocols, none of the protocols yielded pure populations of ECs in our experiment and there was contamination with fibroblast/myofibroblast cells (CK−CD90+CD45−) (data not shown) [23], leukocytes, and other uncharacterized cell populations. A 50% percoll solution has also been tested but no significant increase in the enrichment of ECs from all different cell isolation protocol (as shown in Figs. 1 & 2) was observed (data not shown). To isolate purified ECs, one has to perform single cell sorting experiment from DTT/EDTA treated ECs that might be suitable for further molecular and cultural experiments. The protein tyrosine phosphatase CD45 is abundantly expressed on all nucleated hematopoietic cells and is an essential protein in normal T and B cell development and antigen receptor signaling [37], [38]. The presence of minimal to low frequencies of CK+CD45+ epithelial cells in intestinal tissues demonstrate that these cells might be a population of progenitor ECs as previously described in bone marrow and airway epithelium from mice and humans [39]. The possibility exists that culturing these CK+CD45+ ECs *in vitro* will allow us to further characterize these progenitor cells both phenotypically and functionally and study their distribution in different tissues including the airway, lung, parenchyma, skin, bone marrow and buffy coat.

Epithelial cells provide a shield of the intestinal mucosa from other mononuclear cells below the epithelium, and express HLA-DR molecules in the normal, noninflammed intestinal mucosa. Our data confirms the earlier reports where expression of HLA-DR in ECs has been shown in both small and large intestines [40], [41]. In human, CD90 is absent in thymocytes however both thymocytes and peripheral T cells in mice are positive for CD90 [42]. We have detected the expression of CD90 in all normal healthy uninfected rhesus intestinal ECs. The exact role of CD90 in intestinal ECs is not clear and needs further study to understand its role in immune regulation. In this study we have also noticed increased expression of CD95 and CD23 on EC that are in agreement with previous human intestinal ECs studies [43]–[45]. In inflammatory intestinal diseases, upregulation of CD23 in association with increased MHC class II molecules may suggest lymphoepithelial interactions resulting in exaggerated antigen presentation [45]–[47]. It is possible that the expression of CD23 by normal ECs may have significance in regulating mucosal immunity by serving as a costimulatory molecule that also warrants further study.

A wide variety of isolation methods for human and animal ECs have been used including purely mechanical procedures [48], calcium–chelating agents [49], chelating agents in combination with enzymatic digestion [50], and combinations of enzyme isolation procedures with mechanical preparation [51]. The first successful isolation of human colonic ECs was performed by short enzymatic digestion [52]. In our experiment, viability of ECs is not affected by chemical or enzymatic treatments like DTT, EDTA or collagenase. However, the collagenase enzymatic treatment has always been preferred for the isolation of RM intestinal LPL [53]–[55].

The most important costimulatory receptor expressed by a majority of T cells is CD28, which interacts with CD80 and CD86 ligands on antigen presenting cells and plays a major role in T cell activation. Our studies have shown the lack of CD80 and CD86 expression on intestinal ECs as previously reported for human colonic ECs [56], [57]. In contrast, normal human nasal, airway and oral ECs are able to express different costimulatory molecules including CD80 and CD86 [58], [59]. Constitutive expression of CD23, CD90, CD95 and MHC class II (HLA-DR) expression by normal intestinal ECs may suggest functional significance in regulating mucosal immunity.

IL-10 bears close resemblance to IFNγ and their receptor complexes also belong to the same cytokine receptor family [60]. The expression of IL-10R on different types of RM mucosal cells is also an understudied area. The vast majority of reports of IL-10R expression are on human leukocytes, natural killer cells, neutrophils, macrophages, monocytes, dendritic cells, and/or B and T cells [61]–[64]. Evidence from experiments with the murine small and large intestine has shown that IL-10 binds to a specific receptor that is constitutively expressed on intestinal ECs [65]. Our study highlights the presence of IL-10R in macaque intestinal ECs for the first time, which generates some more questions about how IL-10 binds to this specific receptor on intestinal ECs and how it may regulate the contribution of ECs to the inflammatory and immune response in the digestive tract.

Despite the lack of CD3 and CD4 expression (data not shown) on ECs, there was increased apoptosis of intestinal ECs in both acute (21days pi) and chronically SIV-infected RMs (Fig. 10). Our data is in agreement with previous publications where SIV induced intestinal cell apoptosis is thought to be the underlying mechanism of the regenerative enteropathy of early infection [66], and for enhanced expression of cell cycle genes regulating mucosal repair and regeneration during primary SIV infection [67], [68]. ICAM-1 has been found to be an important adhesion molecule that may facilitate the binding of infected cells to the ECs [69]–[73]. Increased ECs apoptosis was detected with significant upregulation of CD54 expression in chronically SIV-infected RMs. Higher ICAM-1 expression in ECs during SIV infection may suggest its possible role in inducing more HIV transmission and transendothelial migration of T lymphocytes and monocytes [74]–[76]. Expression of ICAM-1 in colonic ECs from patients with colonic carcinoma and inflammatory bowel disease was increased by two fold compared to normal colonic ECs [29]. On the contrary, increased ICAM-1 expression in ECs has also been suggested to result in increased neutrophil adherence to those cells and induce innate defense against bacterial infection [77] suggests a protective mechanism against microbial translocation. SIV infection induces higher ICAM-1 expression on ECs compared to CD45+ leukocytes. In chronically SIV-infected RMs, there was increased HLA-DR expression on both ECs and CD45+ leukocytes. Our observation supports the recent reports describing increased HLA-DR transcripts in gut biopsies of HIV infected patients [78]. The current data supports the notion of immune activation where ECs play a major role by upregulating ICAM-1 and HLA-DR expression that may recruit more inflammatory and cytokine producing cells compared to CD45+ leukocytes. It may also be possible that increased inflammation and cytokine influx in intestinal lamina propria upregulates ICAM-1 expression and apoptosis in ECs.

In summary, either DTT or EDTA chemical treatment yields increased percentages of intestinal ECs compared to other protocols tested. Furthermore, normal healthy rhesus ECs did not express CD80 or CD86 costimulatory molecules. However, they did express CD23 and HLA-DR suggesting they may play a role in antigen presentation, which needs further verification. Combined these results are consistent with the interpretation that ECs are highly activated cells and capable of modulating immune responses. Interestingly, a small percentage of CK+CD45+ ECs in intestinal ECs were always present in normal intestinal mucosa, which invites further exploration to discover the significance of these potential “stem cells” and to define their role in restoration of EC damage and SIV pathogenesis. Early apoptosis and increased expression of ICAM-1 and HLA-DR in ECs in SIV infected RMs are thought to be the key features of intestinal enteropathy that is also associated with local inflammation, and increased gut permeability. The immunoregulatory cells and their cytokines that might play an important role in regulating ECs apoptosis and their activation are the subject of our future studies. The functional implication of CD23, IL-10R and CD90 receptors in the pathophysiological mechanisms of HIV/SIV gut enteropathies, clearly deserves further investigation.

## Materials and Methods

### Ethics statement

Approval for all veterinary procedures in this study was obtained from the Tulane University Animal Care and Use Committee (Protocols #3567 & 3568), Animal Welfare Assurance A-4499-01. All the animals in this project were housed at the Tulane National Primate Research Center (TNPRC) and under the full care of TNPRC veterinarians in accordance with the standards incorporated in the Guide to the Care and Use of Laboratory Animals (NIH) 78-23 (Revised, 1996). All veterinary procedures were performed only with sedated animals. Animal welfare and steps were taken to ameliorate suffering in accordance with the recommendations of the Weatherall report, “The use of non-human primates in research”.

### Animals, and tissue sampling

Sixteen Indian RMs (*Macaca mulatta*) either male or female between 1.9–9.9 years of age, which were negative for HIV-2, SIV, type D retrovirus and STLV-1 infection were used in this study. Six RMs were infected either through intravenous or intravaginal route with 300–500 TCID_50_ SIV_MAC_251. One SIV infected animal was euthanized at 21 days (acute) post infection and five SIV infected animals were euthanized within 167–401 days (chronic) post infection. The remaining 10 macaques were SIV uninfected normal controls. Intestines (jejunum and/or colon) and blood were collected from all animals at the time of necropsy for phenotyping experiments.

### Immunofluorescence and immunoperoxidase staining

Two to three small fresh pieces of colon and jejunum collected from necropsied animals were fixed in 2% paraformaldehyde and cryopreserved in 30% sucrose (Sigma, St. Louis, MO) in PBS, and frozen in cryomolds containing OCT compound (Sakura Fintek, Inc, Torrance, CA). Tissue sections of 8 µm thick were processed for immunofluorescence staining as described previously [79], [80]. Briefly, sections were stained sequentially for 2–3 colors by incubating for 1 h with the primary antibody and then for 30 min with Alexa Flour 488-conjugated secondary antibodies (diluted 1∶1000, Invitrogen). Similarly, the slides were stained again with another primary antibody followed by Alexa Flour 568-conjugated secondary antibodies (dilution 1∶1000, Invitrogen). Nuclear staining was performed with anti-nuclear ToPro3 DNA antibodies (1 µM; Invitrogen). Tissue sections were stained with one or combinations of primary antibodies specified for CD11c (3.9, BioLegend), CD45 (DO58-1283, BDBiosciences), CD54 (LB-2, BDBiosciences), IL-10R (3F9, Biolegend), HLA-DR (LN3, eBiosciences), anti-human macrophage monoclonal antibody (Ham56, Dako), Active caspase-3 (polyclonal antibody, Abcam), human epithelial antigen (polyclonal rabbit anti-cytokeratin, Dako) or cytokeratin-large spectrum monoclonal antibody (KL-1, Beckman Coulter). All antibodies cross react with RMs. Stained tissue sections were mounted using prolong antifade medium (Invitrogen). Imaging was performed with a TCS SP2 True confocal laser scanning microscope (Leica, Wetzlar, Germany) equipped with three lasers. Negative controls were performed by omitting either the primary antibody or using isotype IgG1 and IgG (H+L) control. NIH Image (version 1.62) and Adobe Photoshop CS5 (Adobe Systems) were used to assign colors to the channels collected.

Immunoperoxidase staining of jejunum and colon ECs were performed in 5 µm thick section. The slides were first deparaffinized and heated at 95°C for 30 min in citrate buffer for epitope retrieval. Slides were allowed to cool and finally rinsed in PBS (pH 7.4). Slides were further incubated with endogenous peroxidase block solution (Dako) followed by biotin blocking system (Dako) according to manufacturer's protocol. Slides were then incubated with cytokeratin-large spectrum monoclonal antibody (KL-1). Biotinylated mouse IgG and Vectastain ABC reagent (VECTASTAIN® ABC kit, Vector Laboratories) were used for detection. Liquid DAB (Dako) were used as chromogen for developing slides.

### Epithelial cells isolation from intestine

Intestinal samples (6–10 cm long pieces of jejunum and colon) were collected in 50 ml tubes with ice-cold calcium and magnesium free HBSS (Fisher Scientific) and immediately transported to the laboratory. Fat, blood vessels, and mesenteric lymph nodes were trimmed. Mucus and gross debris were quickly removed by covering the specimen with dry paper towels. Specimens were further washed twice in cold PBS (pH 7.4). Intestinal samples were cut into small pieces (approximately 0.5–1 cm^2^).

All isolation protocols were performed on tissues from necropsied animals to maintain consistency of protocols compared to biopsy protocols where techniques may result in different proportions of ECs in samples. In brief, tissues were treated in 2 major ways. In one technique, the intestinal EC were separated from intestinal pieces by incubating 0.5–1 cm^2^ pieces of tissue in Dithriothreitol (0.15%, DTT, EMD Chemicals) (Fig. 1) [21] followed by EDTA with shaking at 37°C. In another protocol, minced tissues were directly treated with EDTA solution (Fig. 2) as reported earlier [53], [81]. Mucus and large debris were removed from the supernatant by filtering through loosely packed glass wool. After epithelial removal, LPL were collected by mincing the remaining tissue into 1–2 mm pieces, followed by digestion in complete RPMI-5 medium containing 5% fetal calf serum (FCS) (RPMI-5) containing 60 units/ml of Type II collagenase (Sigma-Aldrich) again with shaking at 37°C. For enrichment of lymphocytes, supernatants of LPLs were centrifuged over discontinuous Percoll (Sigma-Aldrich) density gradients followed by washing with PBS [53], [81]. All isolated cells were washed twice and resuspended in complete RPMI-10 medium containing 10% FCS before staining. All cells were >90% viable by trypan blue dye exclusion method.

### Immunofluorescent staining and flow cytometric analysis

For flow cytometry staining, cells were adjusted to 10^7^ cells/ml and 100 µl aliquots or 100 µl of whole blood samples were incubated with appropriately diluted concentrations of antibodies for 30 min at 4°C. Whole blood samples from normal RMs were used for fluorochrome compensations. Whole blood cells were then lysed using a whole blood lysis protocol as previously described [81]. Stained cells were then washed once with PBS and fixed with 1× BD stabilizing fixative buffer (BD Biosciences). Cells were kept protected from light at 4°C and acquisition was performed within 24 hrs of staining. Cells isolated from intestinal tissues were stained and processed similar to blood tissues with the omission of the whole blood lysing technique [81]. Polychromatic (6–10 parameter) flow cytometric acquisition was performed on a Becton Dickinson LSR II instrument with three lasers (488 nm blue laser, 633 nm red laser and 407 violet laser) using FITC, PE, PE-Texas Red, PE-Cy5, APC, APC-Cy7, Pacific Blue, and Amcyan as the available fluorochrome parameters. Anti-CD3 (SP32-2), CD23 (M-L233), CD27 (M-T271), CD54 (LB-2), CD80 (L307.4), CD86 (2331), CD90 (5E10), CD95 (DX2) and HLA-DR (L243) MAbs were obtained from BD Biosciences. CD45 (MB4-6D6) and IL-10R (3F9) were obtained from Miltenyi Biotec and Biolegend respectively. All antibodies are cross reactive to RMs as reported earlier [26], [53], [54], [81]–[85]. To detect EC, intracellular staining was performed by using anti-epithelial antigen (Ber-EP4 from Dako) and cytokeratin-large spectrum MAb (KL1, from Beckman Coulter) as reported earlier [81]. All cells were first gated on live cells where live/dead staining was performed using aqua fluorescent reactive dye live/dead stain kit (Invitrogen). At least 100,000 events were collected from each sample by gating on live cells and data were analyzed using FlowJo software (TreeStar Inc.) version 9.1.

### Statistics

Graphical presentation and statistical analysis of the data were performed using GraphPad Prism (Version 5.0d, GraphPad software, SanDiego, CA). Results between experimental groups were compared using a one-way ANOVA and nonparametric Mann-Whitney t test. Those p values<0.05 were considered statistically significant.
